# Online Remote Behavioural Intervention for Tics (ORBIT-UK): protocol of a single cohort usability study

**DOI:** 10.1136/bmjopen-2025-110121

**Published:** 2026-01-07

**Authors:** Olivia Hastings, Beverley J Brown, Kelly-Marie Prentice, Camilla May Babbage, E Bethan Davies, Joseph Kilgariff, Tara Murphy, Glenn McGarry, Boliang Guo, Chris Greenhalgh, Chris Hollis, Charlotte Lucy Hall

**Affiliations:** 1 Institute of Mental Health, Mental Health & Clinical Neurosciences, School of Medicine, University of Nottingham, Nottingham, UK; 2 NIHR MindTech HealthTech Research Centre, Institute of Mental Health, University of Nottingham, Nottingham, England, UK; 3 Institute of Mental Health, Mental Health & Clinical Neurosciences, University of Nottingham, Nottingham, UK; 4 NIHR MindTech HealthTech Research Centre, University of Nottingham, Nottingham, UK; 5 NIHR MindTech Medtech Co-operative, Institute of Mental Health, University of Nottingham Faculty of Medicine and Health Sciences, Nottingham, UK; 6 Queen’s Medical Centre, Nottingham, Nottingham, UK; 7 University College London Great Ormond Street Institute of Child Health, London, UK; 8 Great Ormond Street Hospital for Children NHS Foundation Trust, London, UK; 9 School of Computer Science, University of Nottingham, Nottingham, UK; 10 Psychiatry, Institute of Mental Health, Nottingham, Nottinghamshire, UK; 11 Division of Psychiatry, Nottinghamshire Healthcare NHS Foundation Trust, Nottingham, England, UK

**Keywords:** Developmental neurology & neurodisability, Digital Technology, Clinical Protocols, Child & adolescent psychiatry, Paediatric neurology, MENTAL HEALTH

## Abstract

**Introduction:**

Tourette syndrome is a common, disabling childhood-onset condition. Exposure and response prevention (ERP) is an effective treatment for tics, yet access remains limited due to a shortage of trained therapists and uneven geographical distribution of services. The ORBIT trial demonstrated that internet-delivered ERP is both clinically and cost-effective, but was developed on a university research platform, not suitable for widescale roll-out. To enable adoption by the National Health Service (NHS) in England, ORBIT has been redeveloped on an NHS compliant platform. This study will evaluate the usability, acceptability and preliminary outcomes of ORBIT on the new platform within an NHS tic disorder service.

**Methods and analysis:**

This single-cohort usability study will recruit 20 children and young people (aged 9–17) with tics and their chosen supporters (parents/carers). Participants will receive a 10-week online ERP intervention supported by trained coaches. Outcomes include uptake, adherence, system usability, satisfaction and clinical measures such as the Yale Global Tic Severity Scale, Parent Tic Questionnaire and Goal-Based Outcomes. Qualitative feedback will be collected via semi-structured exit interviews. Usability metrics and adverse events will be monitored throughout.

**Ethics and dissemination:**

The study has received ethical approval from North West Greater Manchester Research Ethics Committee (ref: 25/NW/0107). The findings from the study will inform future NHS adoption. The results will be submitted for publication in peer-reviewed journals.

**Trial registration number:**

ISRCTN82718960. Registered 10 July 2025. https://doi.org/10.1186/ISRCTN82718960

**STRENGTHS AND LIMITATIONS OF THIS STUDY**

- Evaluates the real-world usability of a previously validated digital intervention on a new technical platform, providing insights into user experience, real-world outcomes and implementation feasibility.
- Provides a practical example of post-trial validation, demonstrating how a clinically effective intervention can be transitioned from an academic research platform to a patient-ready product.
- Patient and public involvement embedded in every stage of the research including platform development.
- The small sample size (n=20) limits generalisability of findings, although it is appropriate for a usability-focused study.
- As a single-cohort study without a comparator, the study does not assess performance against other platforms or delivery methods.

## Introduction

 Tourette syndrome (TS) is a common, childhood onset condition that affects up to 1% of children and young people (CYP) and is associated with high levels of comorbidity, significant distress, psychological impairment and reduced quality of life.^1^ Most patients with TS experience co-occurring conditions such as attention deficit hyperactivity disorder, obsessive-compulsive disorder, depression, anxiety and self-injurious behaviour,^2^ making interventions complex.

Evidence-based interventions for the treatment of tics in CYP include pharmacological treatment and behaviour therapy (BT), such as exposure and response prevention (ERP) or habit reversal training (HRT).^3^,^5^ The effectiveness of BT in reducing tics is well established.^1^ A recent randomised controlled trial demonstrated that ERP was as effective as medication in reducing tic severity, with superior maintenance of treatment gains at 3-month follow-up and greater treatment adherence, likely due to fewer adverse effects.^6^ In England, the National Institute for Health and Care Excellence (NICE) set clinical guidelines for the delivery of care across different conditions. Currently, while no NICE guidelines exist for the management of tics in CYP, both European clinical guidelines^4^ and a Health Technology Assessment evidence synthesis^7^ recommend offering BT as a first-line intervention in a stepped-care approach.

Despite these recommendations, only 1 in 5 CYP with TS in the UK are currently able to access BT for tics.^8^ Moreover, many of those who do access treatment often receive fewer than half the recommended number of sessions.^8^ A critical shortage of trained therapists, coupled with uneven service distribution across the UK, presents a major barrier to equitable access to therapy.^8^

Digital health interventions offer a promising solution to close this gap in treatment provision. A recent randomised controlled trial, ‘Online Remote Behavioural Intervention for Tics’ (ORBIT) trial, found that online delivered, therapist supported ERP is clinically and cost effective.^9 10^ ORBIT led to significantly greater reductions in tic severity (16%) compared to an active control condition (psychoeducation, 6%)^10^; an effect that sustained throughout 6-, 12- and 18-month follow-ups.^11^ A process evaluation confirmed that the intervention was implemented with high fidelity and was highly acceptable to participants.^12 13^ Engagement was rated as excellent, with participants completing an average of 7.5/10 chapters and 88.4% of participants reaching the predefined effective treatment threshold of 4 chapters.

A full economic evaluation found that at 18 months, online ERP had a 79% probability of being cost-effective compared with psychoeducation, at a £30 000 per quality-adjusted life year (QALY) threshold.^7^ While ORBIT represents an additional intervention, health economic analysis suggests its integration into the NHS could reduce overall costs by decreasing reliance on less effective services.^14^ Additionally, the therapist-guided format only required an average of 2.5 hours total clinician time per participant, versus 9–10 hours required for face-to-face BT.^7^

Following this trial, NICE has conducted an early value assessment (EVA) of ORBIT and provisionally approved its use in clinical settings while further evidence is generated.^15^ However, the ORBIT intervention was delivered using the Swedish ‘BiP-TiC’ platform^16^ and is currently not available as a patient-ready product delivered on an NHS compliant platform in the UK. To enable future NHS adoption, it is necessary to re-develop the intervention on a UK-based, NHS-compliant platform and evaluate its usability. As such, this study aims to assess the usability, uptake, acceptability and preliminary outcomes of the ORBIT-UK intervention within an NHS tic disorder service. The intervention will be redeveloped from the original ORBIT trial into a clinical product compatible with NHS systems and commissioning pathways, supporting its future adoption and scale-up. This study will provide a practical example of post-trial validation, demonstrating how an effective clinical trial intervention can be transitioned from an academic research platform to a new technical environment while maintaining core components determining clinical effectiveness within a user-centred evaluation.

### Objectives

The objectives for this study are:

1. To test the acceptability of the ORBIT-UK platform: a coach-guided, remotely delivered behavioural intervention for tics in young people, and the feasibility of the ORBIT-UK service pathway in the NHS. Acceptability and feasibility will be measured through achievement of recruitment and adherence targets. The recruitment target for this study is n=20. To ensure that the findings align with the ORBIT trial, we will benchmark against the following criteria, with a breakdown of targets detailed in table 1.

1. To test the acceptability of the ORBIT-UK platform: a coach-guided, remotely delivered behavioural intervention for tics in young people, and the feasibility of the ORBIT-UK service pathway in the NHS. Acceptability and feasibility will be measured through achievement of recruitment and adherence targets. The recruitment target for this study is n=20. To ensure that the findings align with the ORBIT trial, we will benchmark against the following criteria, with a breakdown of targets detailed in table 1.

**Table 1:** Benchmark criteria for acceptability and feasibility

Criteria	Good (green)	Poor (red)
Recruitment	20 accept out of up to 27 offered ORBIT and eligible (expected for 85% of random samples)	20 accept out of 30 or more referred (expected in only 5% of random samples)
Adherence to intervention (completion of first 4 chapters)	15 or more complete at least the first 4 chapters (expected for 80% of random samples)	Fewer than 12 do not complete at least the first 4 chapters (expected in only 1% of random samples)

These boundary criteria are based on quantiles of a binomial distribution or Bernoulli trials with probability of 0.8 for uptake and for adherence. Percentiles are chosen to best align with subject numbers and to represent poor or good under the 0.8 probability assumption.

2. To gather feedback from young people, supporters, coaches (renamed from ‘therapist’ for ORBIT-UK) and clinicians on the acceptability and usability of both the platform and the service pathway.
3. To identify strengths and weaknesses of the platform through usability testing.
4. To present the change from baseline to post-intervention clinical outcomes to explore symptom changes following completion of the ORBIT-UK platform.
5. To investigate the safety of the ORBIT-UK platform.

## Methods and analysis

### Study design

This study is a 10–12 week, single cohort usability study for CYP with tics. Participants will receive 10 weeks of online, remotely delivered, coach-supported behavioural therapy for tics. The study endpoint will be 3 months post completion of baseline measures, when participants will complete the post-intervention measures within 2 weeks of finishing the intervention. The overall study duration will be 13 months (from the first participant enrolled to last post-intervention assessment). The planned recruitment start date is 1 January 2025, with an estimated end date of 21 September 2026. Overall study completion will be 3 March 2027, and findings will be published within 1 year of data collection. See online supplemental file 1 for an overview of the participant pathway through the study.

### Setting

The intervention will be delivered within a single Tic Disorder Service. Patients are referred into this service by community paediatricians or child mental health services.

### Recruitment and Participants

Eligible participants will be recruited from within the Tic Disorder Service and offered the ORBIT intervention. The initial approach will be from a member of the patient’s usual care team within the Tic Disorder Service. Patients (young people) will be provided with an age-appropriate participant information sheet. Parent/carers will be provided with participant information sheets detailing the clinical study and ORBIT intervention.

#### Inclusion criteria

1. Aged 9 to 17 years: patient confirmed at screening.
2. Suspected or confirmed TS/chronic tic disorder.
3. Including moderate/severe tics: score >15 on the Yale Global Tic Severity Scale (YGTSS) Total Tic Severity Score (TTSS); TTSS score >10 if motor or vocal tics only: Coach will assess this at initial assessment via video conference.
4. Competent to provide written, informed consent (parental consent for child aged <16): referring clinician confirms at screening appointment.
5. Broadband internet access and regular PC/laptop/Mac/mobile device user, with mobile phone SMS: patient confirmed at screening.
6. Clinical suitability for ORBIT-UK confirmed by referrer from the patients’ usual care team. Clinical suitability refers to the clinician’s judgement that ORBIT is an appropriate therapeutic option, based on clinical presentation, motivation to engage and capacity to undertake the intervention.
7. A parent/carer/legal guardian aged 18+ who is willing and able to act as a supporter.

#### Exclusion criteria

1. Functional tics as primary presentation.
2. Moderate/severe intellectual disability: confirmed through qualitative judgement of the referrer from the patients’ usual care team.
3. Immediate risk to self or others: confirmed through referrer from the patients’ usual care team.
4. Parent or child not able to speak or read/write English: patient confirmed through screening by the referring clinician.

#### Additional Eligibility Criteria (Supporters, Clinicians and Coaches)

1. Supporters: A supporter can be a parent, caregiver or legal guardian who is 18 years or older and has the capacity to support CYP through the intervention.
2. Clinicians: Only clinicians who appear on the ORBIT site delegation log will be eligible to be approached to be interviewed.
3. Coaches: Only coaches who appear on the ORBIT site delegation log will be eligible to be approached to be interviewed.

### Screening

The healthcare team in the Tic Disorder Service will approach suitable participants and provide them with participant information sheets. Informed consent will be taken at this screening appointment by the usual treating clinician, further details about which are listed under ‘ethics and dissemination’ and consent forms can be viewed in online supplemental file 2. After obtaining informed consent from young people and their supporters, the clinician will log into the ORBIT platform and complete the automated pre-screening filter with details about the young person to confirm eligibility. If successful in passing the filter, the clinician will go on to complete the main screening assessment which includes demographic information of the participant and their supporter. The screening measures will sit securely within the ORBIT platform. The clinician will assist in scheduling a baseline assessment with an assigned ORBIT Coach.

#### Remote Baseline Assessment

During the baseline assessment, CYP and their supporters will virtually meet their ORBIT coach through Microsoft Teams, who will introduce the ORBIT platform to them. Coaches will provide CYP and their supporters with logins and set a start date for intervention (typically within 24–48 hours of the baseline appointment). During this videocall, the ORBIT-UK Coach will complete the YGTSS^17^ with the YP. Coaches will also assist the YP in setting 1–3 Goal Based Outcomes^18^ (GBOs) based on their choice.

#### Intervention

ORBIT is a web-based intervention for CYP with tics, based on ERP. The platform has been developed by Blum Health (https://blumtechgroup.com/) and is designed with age-appropriate visuals, animations and interactive scripts. The intervention comprises 10 online chapters for CYP and parallel chapters for a designated supporter and is designed to last 10–12 weeks.

Each chapter takes approximately 30–45 min to complete and includes psychoeducation, interactive exercises and ERP skills practice.^7 19^

Participants will receive support, guidance and feedback from a coach via in-platform messaging. The primary Coach will be employed by Nottinghamshire Healthcare Foundation Trust (NHCFT) (Band 5 Assistant Psychologist), and the secondary Coach, a University of Nottingham research assistant, will work within NHCFT under an honorary contract. Coaches will be trained in administering the YGTSS, good clinical practice (GCP) and supervised by the NHCFT clinical team. Neither has prior experience delivering behavioural therapy for tic disorders.

Participants typically have contact with their coach several times per week. Both CYP and supporters have separate logins and equal access to the Coach. Optional downloadable resources include a credit and reward scheme, behaviour tracking tools, teacher information handouts and strategies for managing difficult situations. These materials were developed in the previous ORBIT trial^9 10^ and are intended to enhance, but not replace, the core intervention.

### Measures and Outcomes

#### Baseline

As above, during the remote baseline appointment, the coach will complete the YGTSS and set GBOs with the young person. This appointment will last approximately 1 hour. The YGTSS is a semi-structured interview focussing on motor and vocal tic frequency, severity and tic-related impairment over the previous week. The YGTSS produces a TTSS (0–50) and an impairment score (0–50), with higher scores indicating greater severity/impairment.

GBOs will also be set at baseline, which are realistic, intervention-specific goals (eg, ‘to have better control over my tics’). Young people will rate their progress on a 0–10 scale at the end of each chapter, where 0 means no progress and 10 means the goal is fully achieved.

Following this appointment, additional baseline measures will also be collected from participants through the ORBIT-UK platform. Participants must log in and self-complete these measures before the agreed intervention start date. Once complete, the first chapter of the intervention will be automatically made available.

Completed by the Supporter:

- The Parent Tic Questionnaire (PTQ)^20^ : The PTQ assesses the number, frequency and intensity of motor and vocal tics in children and adolescents with tics from their parent/carer’s perspective. The PTQ takes under 10 min to complete.

Completed by the Young Person:

- The Child and Adolescent Gilles de la Tourette Syndrome-Quality of Life Scale (C&A GTS-QoL).^21^ The C&A GTS-QoL is a measure of health-related quality of life specifically designed for young people with TS to assess psychological, physical, obsessive-compulsive and cognitive domains of quality of life over the last 4 weeks. Scores are calculated for each domain and summed to provide a total score, with higher scores indicating greater impairment in quality of life. This takes approximately 5 min to complete.

#### During the intervention

At the end of each chapter, participants will complete the following measures:

- A review of progress towards GBOs.
- A chapter usability rating.
- On-going adverse event (AE) reporting, if necessary, through an in-built ‘Report AE’ function.

#### Mid-intervention (week 5)

Supporters will complete the PTQ to give an indication of how the Young Person is coping with their tics.

#### End of the intervention

The end of intervention has been defined as Week 8, based on findings from the previous ORBIT trial that showed most participants reached this stage, and all therapeutic content will have been delivered. At this stage, participants will complete a series of outcome measures including:

Completed by the Supporter:

- PTQ
- System Usability Scale (SUS).^22^ The SUS is a widely used, 10-item Likert questionnaire designed to assess the usability of a website or platform. This takes under 5 min to complete.
- ORBIT Satisfaction Questionnaire.^10^ This is an 8-item measure, with responses rated on a 5-point Likert scale and summed to produce a total satisfaction score, where higher scores reflect greater satisfaction with the intervention. This takes under 5 min to complete.

Completed by the Young Person:

- GTS-QoL.
- SUS.
- ORBIT Satisfaction Questionnaire.

Once a participant has completed all chapters, their coach will schedule a Teams call to administer the YGTSS with the Young Person (Weeks 10–12). After this call, the Coach will fill out the Clinical Global Impressions Improvement Scale to measure global symptom improvement.^23^ This is a 7-point Likert scale that allows the Coach to assess improvement from baseline. Please see table 2 for an overview of all study outcome measures and timepoints.

**Table 2:** Schedule of outcome measures

Months post-enrolment	0	0	0–3	1	2	3	Completed by	Mode of delivery
Time point	Screening	Baseline (Pre-treatment)	Per-Chapter/on-going	Mid-treatment (5 weeks)	8 weeks	10–12 weeks (treatment end)		
Consent	X						S/YP	REDCap
Automated pre-screening filter (Part A)	X						CL	ORBIT platform
Referral form and demographics (Part B)	X						CL	ORBIT platform
PTQ		X		X	X		S	ORBIT platform
YGTSS		X				X	CO/YP	Videocall (Teams)
CGI-I						X	CO	ORBIT platform
GBO		X	X	X	X	X	YP	ORBIT platform
Chapter rating			X	X	X	X	S/YP	ORBIT platform
C&A GTS-QOL		X			X		YP	ORBIT platform
SUS					X		S/YP	ORBIT platform
Adverse effects/side effects		X	X	X	X	X	S/YP	ORBIT platform
ORBIT satisfaction					X		S/YP	ORBIT platform
Exit interview						X	S/YP/CL/CO	Videocall (Teams)

C&A-GTS-QOL, The child and adolescent version of the Gilles de la Tourette Syndrome Quality of Life Scale; CGI-I, The Clinical Global Impressions Improvement Scale; CL, clinician; CO, coach; GBO, Goal Based Outcomes; ORBIT, Online Remote Behavioural Intervention for Tics trial; PTQ, Parent Tic Questionnaire; S, supporter; SUS, System Usability Scale; YGTSS, Yale Global Tic Severity Scale; YP, young person.

#### Exit interview

At the end of the intervention, both young people and their supporters will be invited to participate in an exit interview with the researcher, which may be face-to-face at the Tic Disorder Clinic or via Teams videoconferencing. The interview is optional, and participants will have indicated whether they wish to be contacted regarding the interview when completing the informed consent form. The interview will last approximately 30 min and is designed to gather qualitative feedback on intervention usability, acceptability and preliminary effectiveness. Interviews will also be conducted with coaches and the coach’s clinical supervisor.

### Agreement with Expert Rater Procedure (YGTSS)

Agreement with an expert rater will be assessed by the trial manager (BJB), who will independently score 10% of YGTSS assessments. BJB will observe coaches completing the YGTSS, and when live observation is not possible, coaches will complete a standardised assessment with BJB acting as a mock participant.

Coaches must score within 15% of BJB’s scores, following the approach described in previous research.^24^ Agreement calculations will be assessed by the statistician. Coaches not meeting the threshold will receive additional training and repeat scoring.

### Sample Size

As an exploratory study, the sample size (n=20) will be used to evaluate the usability of the ORBIT-UK platform. This is in line with observed sample sizes in other feasibility and pilot studies in the UK Clinical Research Network Database.^25^

### Patient and Public Involvement

The National Institute for Health and Care Research (NIHR) INVOLVE, a national advisory group supporting active public involvement in health and social care research, define patient and public involvement (PPI) as “research being carried out ‘with’ or ‘by’ members of the public rather than ‘to’, ‘about’ or ‘for’ them”.^26^ Involving people with lived experience of tics is essential for developing a relevant and accessible intervention that is shaped by patient and parent/carer perspectives.^27 28^

The NIHR recommends involving PPI from the very early stages in the research cycle, as was the case for ORBIT-UK.^29^ ORBIT-UK has had a PPI member included in the project from the point of grant development with the production of the lay summary and advice given on the project aims, ensuring the use of patient-friendly language in the documentation and relevance of the project to the community it intends to impact. The grant was also informed by insights from the previous PPI group from the ORBIT trial in the selection of outcome measures, as well as the decision to rename ‘therapists’ to ‘coaches’.

In addition, our PPI member was also integral at highlighting the importance of representation in the PPI panel, advocating for the involvement of both parents and children in our PPI panel and suggesting all PPI meetings are scheduled outside of school and work hours to allow for greater representation of family perspectives.

This led to the development of a panel of 10 parents and adults with TS who will meet monthly, and a panel of 5 young people who will meet quarterly throughout the study. Meetings will be held separately for young people and parents/adults to maximise rapport and accessibility. One parent/child dyad will be invited to participate in Research Steering Group (RSG) meetings.

The PPI panel will contribute to six key areas:

- Co-designing the ORBIT-UK platform.
- User testing.
- Reviewing participant documents and protocols.
- Developing lay-appropriate interview guides.
- Selecting outcome measures.
- Supporting dissemination through events and publications.

The PPI panel will help disseminate this research by attending project events, such as at Commissioners Events, and through co-authorship on conference posters. A bi-monthly ORBIT-UK newsletter keeps PPI members informed of study progress. The PPI panel will receive payment for all PPI activities in line with NIHR payment guidelines.^30^ To keep a record of how PPI has informed the current study and for transparency in reporting, the research team will use the Guidance for Reporting Involvement of Patients and the Public (version 2) (GRIPP2) checklist.^31^

### Data Management and Analysis

All data will be collected, stored and managed in accordance with the UK Data Protection Act 2018 and GCP. Electronic data will be stored securely on a password-protected database with access restricted via multi-factor authentication. Identifiable data will be removed prior to analysis, and a unique study ID will be assigned to each participant. Data will be anonymised for publication and shared only with authorised personnel. A Data Management Plan and Data Protection Impact Assessment are in place.

#### Quantitative Analysis

This study evaluates the usability of the ORBIT-UK platform; therefore, analyses of clinical effectiveness are not planned, as ORBIT’s efficacy and cost effectiveness have already been demonstrated.^10 11^

Descriptive statistics will be used to summarise uptake, adherence and outcome measures across time points. Uptake and adherence will be classified as good or poor (table 1). Outcomes will be summarised across time points using means and SD for normally distributed variables, medians (IQR) for skewed variables and frequencies (%) for categorical variables. Changes in outcomes from baseline to follow-up will be quantified by regression models (linear or non-linear, as appropriate). Non-independence due to repeated measures will be addressed using multilevel models or regression with robust standard errors.

As the study does not test treatment effectiveness, missingness will be summarised but not imputed for usability and adherence outcomes; analyses will use observed data only. For exploratory clinical outcomes, sensitivity analyses may be considered using appropriate imputation methods.

#### Qualitative Analysis

Exit interviews will be transcribed using University of Nottingham approved transcription software and coding and theming of the data will be completed using NVivo and spreadsheet software such as Microsoft Excel ^32 33^ .

### Monitoring

#### Management and Oversight

The Chief Investigator is responsible for overall study management and will act as the data custodian. Day-to-day operations will be managed by a dedicated Trial Manager (TM) in collaboration with the Senior Project Manager, who will coordinate delivery to time and target, maintain the study risk register and report to stakeholders. Strategic oversight will be provided by a multidisciplinary RSG, comprising clinicians, academics, third-sector partners and health economists, which will meet biannually to ensure ethical conduct and monitor progress and safety. Study governance is further supported by the Project Management Group and Intellectual Property Management Group, both meeting quarterly to address commercialisation readiness, risk mitigation and progress.

#### Adverse Events

There are no anticipated related serious adverse events for this study. Adverse events will be recorded via in-built adverse events feature within the ORBIT-UK platform, by coaches and by participants themselves. Participants will be informed of what constitutes an adverse event during their baseline appointment. Coaches will notify the research team of any adverse events that are reported and escalate any reports of concern to their clinical supervisor, who will determine whether the AE meets the criteria for a serious adverse event (SAE). All SAEs will be reported by the site clinician to the CI and unexpected SAEs will be reported to the Research Ethics Committee (REC) within 7 days.

### Ethics and Dissemination

The study received ethical and Health Research Authority approval from North West Greater Manchester Central Research Ethics Committee on 29 April 2025 (ref: 25/NW/0107: protocol v1.0, 13 March 2025). Local approval has been granted by the participating Trust. The study is sponsored by the University of Nottingham. Neither the sponsor nor the funders will be involved in the analysis of the study data or report writing. Data will be available for inspection by the ethics committee on request. Changes to the protocol will be communicated to the ethics committee, sponsor and funders by the TM. The process for obtaining informed consent or assent will be in accordance with ethical guidance and GCP. Where the young person is 16 years and over, written consent will be required from both the parent and young person. Where the young person is under 16 years, written parental consent will be required, alongside the young person’s assent. In the event of any conflict between the parent and child, the child will not enter the study. Participant confidentiality will be ensured by anonymising outcome data by using identification numbers in data analysis. At the end of the study, participants will have access to the intervention for 1 year after starting treatment, although this will be without the coach support.

The findings will be published in peer-reviewed journals, presented at relevant conferences and disseminated to the public via lay summaries co-created with our PPI group. All outputs will be authored by the research team and will not involve professional writers. Access to the full protocol is available on request to the corresponding author. The chief investigator declares no financial or competing interests.

## Discussion

This study will provide a practical example of post-trial validation, demonstrating how a clinically and cost-effective intervention can transition from an academic research platform to a patient-ready product. Building on the success of the ORBIT trial,^10^ which demonstrated significant and sustained reductions in tic severity, this study will evaluate the usability of the ORBIT platform in routine clinical care.

The development of ORBIT on an NHS compliant platform ensures technical alignment with NHS infrastructure and commissioning requirements. Embedding ORBIT within an existing NHS Tic Disorder Service will provide a clear example for broader NHS adoption. This work aligns with NHS priorities around digital transformation and early intervention in mental health. ORBIT has already undergone a NICE EVA, receiving provisional approval for clinical use while additional evidence is generated. The outcomes of this study will directly inform NICE’s ongoing evaluation and guide the development of commissioning pathways for national implementation.

In summary, this study has the potential to significantly improve access to effective treatment for tics, by demonstrating how ORBIT can be delivered within the NHS. The findings will support its integration into national care pathways, offering a scalable solution to a long-standing gap in tic disorder services for young people.

## Supplementary material

10.1136/bmjopen-2025-110121 online supplemental file 1

10.1136/bmjopen-2025-110121 online supplemental file 2
