# Supplementary figures and images for: Online Remote Behavioural Intervention for Tics (ORBIT-UK): protocol of a single cohort usability study
Source: BMJ Open. 2026 Jan 7;16(1):e110121. doi: 10.1136/bmjopen-2025-110121 (PMC12781982; doi:10.1136/bmjopen-2025-110121)

# ORBIT-UK Participant Pathway

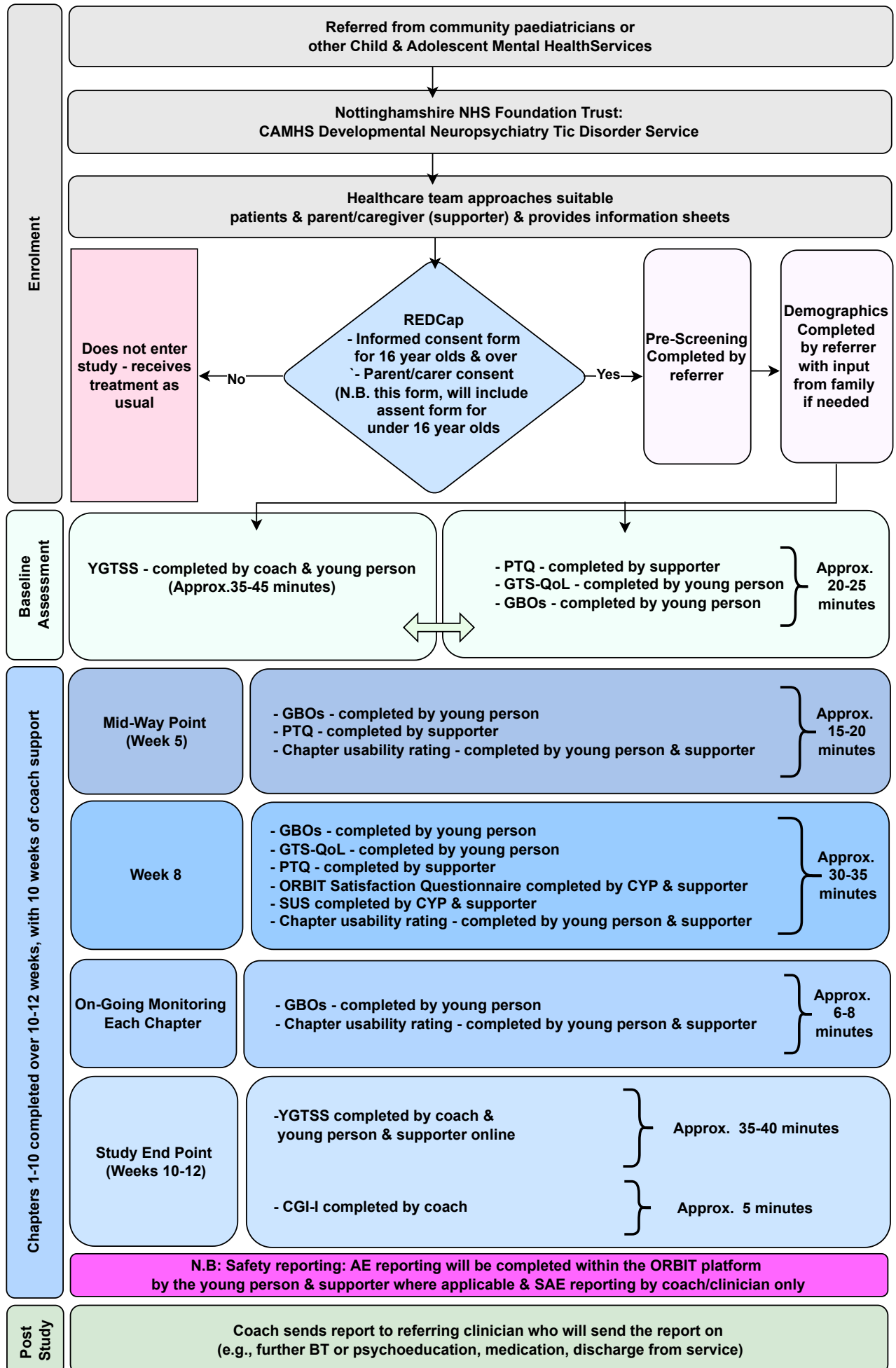

Supplement: online supplemental file 1 [file bmjopen-16-1-s001.pdf]
