# Supplementary material for: Online Remote Behavioural Intervention for Tics (ORBIT-UK): protocol of a single cohort usability study
Source: BMJ Open. 2026 Jan 7;16(1):e110121. doi: 10.1136/bmjopen-2025-110121 (PMC12781982; doi:10.1136/bmjopen-2025-110121)
Supplement: online supplemental file 2 [file bmjopen-16-1-s002.pdf]

FUNDED BY NIHR | National Institute for Health and Care Research

University of Nottingham
UK | CHINA | MALAYSIA

ORBIT
Online Remote Behavioural Intervention for Tics

(Form to be printed on local headed paper)

ORBIT-UK CONSENT FORM FOR PARENT/CARER

Final Version 1.1 12-Sept-2025

Title of Study: Online Remote Behavioural Intervention for Tics (ORBIT-UK)

IRAS Project ID: 351072

Participant ID:

Please initial box

1. I confirm that I have read and understand the information sheet version number XXX dated XXX for the above study and have had the opportunity to ask questions. ☐

2. I understand that my participation is voluntary and that I am free to withdraw at any time, without giving any reason, and without my legal rights being affected. I understand that should I withdraw then the information collected so far cannot be erased and that this information may still be used in the project analysis. ☐

3. I understand that relevant sections of my data collected in the study may be looked at by authorised individuals from the University of Nottingham, the research group and regulatory authorities where it is relevant to our taking part in this study. I give permission for these individuals to have access to these records and to collect, store, analyse and publish information obtained from my participation in this study. I understand that my personal details will be kept confidential. ☐

(Form to be printed on local headed paper)

4. I agree that the information gathered about me can be stored by the University of Nottingham for possible use in future studies. I understand that some of these studies may be carried out by researchers other than the current team who ran the first study, including researchers working for commercial companies. Any data used will be anonymised, and I will not be identified in anyway. ☐

5. I would like to receive a summary of the results at the end of the study and agree for my contact details to be retained and used for this purpose. Yes ☐ No ☐

6. I consent to being contacted and interviewed by a member of the research team. Yes ☐ No ☐

7. I understand the interview will be recorded and anonymous direct quotes from these interviews may be used in study reports ***(N.B. please note that this will be an online consent form, and that this option will only appear if option 6 is marked as a 'yes').*** Yes ☐ No ☐

8. I agree (named above) to take part in the above study. ☐

Name of Parent	Date	Signature

2 copies: 1 for participant, 1 for the project notes

(Form to be printed on local headed paper)

______________________	____________	__________________
Name of Person taking consent	Date	Signature

2 copies: 1 for participant, 1 for the project notes

(Form to be printed on local headed paper)

ORBIT-UK CONSENT FORM FOR PARENTS CYP UNDER 16 YEARS

Final Version 1.1 12-Sept-2025

Title of Study: Online Remote Behavioural Intervention for Tics (ORBIT-UK)

IRAS Project ID: 351072

Participant ID: **Please initial box**

9. I confirm that I have read and understand the information sheet version number XXX dated XXX for the above study and have had the opportunity to ask questions. ☐

10. I understand that my child's participation is voluntary and that they are free to withdraw at any time, without giving any reason, and without their medical care or legal rights being affected. I understand that should they withdraw then the information collected so far cannot be erased and that this information may still be used in the project analysis. ☐

2 copies: 1 for participant, 1 for the project notes

(Form to be printed on local headed paper)

11. I understand that relevant sections of my child's medical notes and data collected in the study may be looked at by authorised individuals from the University of Nottingham, the research group and regulatory authorities where it is relevant to our taking part in this study. I give permission for these individuals to have access to these records and to collect, store, analyse and publish information obtained from my child's participation in this study. I understand that my child's personal details will be kept confidential. ☐

12. I agree that the information gathered about my child can be stored by the University of Nottingham, for possible use in future studies. I understand that some of these studies may be carried out by researchers other than the current team who ran the first study, including researchers working for commercial companies. Any data used will be anonymised, and my child will not be identified in anyway. ☐

13. I would like to receive a summary of the results at the end of the study and agree for my contact details to be retained and used for this purpose. Yes ☐ No ☐

14. I consent to being contacted so that my child can be interviewed by a member of the research team. Yes ☐ No ☐

15. I understand the interview will be recorded and anonymous direct quotes from these interviews may be used in study reports ***(N.B. please note that this will be an online consent form, and that this option will only appear if option 6 is marked as a 'yes').*** Yes ☐ No ☐

8. I agree for my child (named above) to take part in the above study. ☐

____________________ ____________ __________________

Name of Parent | Date | Signature

2 copies: 1 for participant, 1 for the project notes

(Form to be printed on local headed paper)

Name of Person taking consent	Date	Signature

(OPTIONAL) Section for children to give assent

I agree to take part in this study

Name of Child (for assent)	Date	Signature

2 copies: 1 for participant, 1 for the project notes

(Form to be printed on local headed paper)

ORBIT-UK CONSENT FORM TO INTERVIEW FOR COACHES AND CLINICIANS

Final Version 1.1 12-Sept-2025

Title of Study: Online Remote Behavioural Intervention for Tics (ORBIT-UK)

IRAS Project ID: 351072

Participant ID: **Please initial box**

1. I confirm that I have read and understand the information sheet version number XXX dated XXX for the above study and have had the opportunity to ask questions. ☐

2. I understand that my participation is voluntary and that I am free to withdraw at any time, without giving any reason, and without my legal rights being affected. I understand that should I withdraw then the information collected so far cannot be erased and that this information may still be used in the project analysis. ☐

2 copies: 1 for participant, 1 for the project notes

(Form to be printed on local headed paper)

3. I understand that relevant data collected may be looked at by authorised individuals from the University of Nottingham, the research group and regulatory authorities where it is relevant to my taking part in this study. I give permission for these individuals to have access to this data, and to collect, store, analyse and publish information obtained from my participation in this study. I understand that my personal details will be kept confidential. ☐

4. I understand that the interview will be recorded and that anonymous direct quotes from the interview may be used in the study reports. ☐

5. I understand that the information collected about me will be used to support other research in the future and may be shared anonymously with other researchers. ☐

6. I would like to receive a summary of the results at the end of the study and agree for my contact details to be retained and used for this purpose. Yes ☐ No ☐

7. I agree to take part in the above study. ☐

(Form to be printed on local headed paper)

Name of Participant	Date	Signature
Name of Person taking consent	Date	Signature

2 copies: 1 for participant, 1 for the project notes

(Form to be printed on local headed paper)

ORBIT-UK CONSENT FORM FOR YOUNG PEOPLE 16 YEARS AND OVER

Final Version 1.1 12-Sept-2025

Title of Study: Online Remote Behavioural Intervention for Tics (ORBIT-UK)

IRAS Project ID: 351072

Participant ID: **Please initial box**

1. I confirm that I have read and understand the information sheet version number XXX dated XXX for the above study and have had the opportunity to ask questions. ☐

2. I understand that my participation is voluntary and that I am free to withdraw at any time, without giving any reason, and without my medical care or legal rights being affected. I understand that should I withdraw then the information collected so far cannot be erased and that this information may still be used in the project analysis. ☐

(Form to be printed on local headed paper)

3. I understand that relevant sections of my medical notes and data collected in the study may be looked at by authorised individuals from the University of Nottingham, the research group and regulatory authorities where it is relevant to my taking part in this study. I give permission for these individuals to have access to these records and to collect, store, analyse and publish information obtained from my participation in this study. I understand that my personal details will be kept confidential. ☐

4. I Consent for storage and use in possible future research. I agree that the information I have given, and the information gathered about me can be stored by the University of Nottingham, for possible use in future studies. I understand that some of these studies may be carried out by researchers other than the current team who ran the first study, including researchers working for commercial companies. Any data used will be anonymised, and I will not be identified in anyway. ☐

5. I would like to receive a summary of the results at the end of the study and agree for my contact details to be retained and used for this purpose. **Yes** ☐ **No** ☐

6. I consent to being contacted and interviewed by a member of the research team. **Yes** ☐ **No** ☐

7. I understand that the interview will be recorded and anonymous direct quotes from these interviews may be used in study reports ***(N.B. please note that this will be an online consent form, and that this option will only appear if option 6 is marked as a 'yes').*** **Yes** ☐ **No** ☐

8. I agree to take part in the above study. ☐

2 copies: 1 for participant, 1 for the project notes

FUNDED BY NIHR | National Institute for Health and Care Research

University of Nottingham UK | CHINA | MALAYSIA

(Form to be printed on local headed paper)

Name of Participant	Date	Signature
Name of Person taking consent	Date	Signature

2 copies: 1 for participant, 1 for the project notes
