# Supplementary material for: Online Remote Behavioural Intervention for Tics (ORBIT-UK): protocol of a single cohort usability study
Source: BMJ Open. 2026 Jan 7;16(1):e110121. doi: 10.1136/bmjopen-2025-110121 (PMC12781982; doi:10.1136/bmjopen-2025-110121)
Supplement: Supplementary file 3 [file bmjopen-2025-110121.reviewer_comments.pdf]

PEER REVIEW HISTORY

BMJ Open publishes all reviews undertaken for accepted manuscripts. Reviewers are asked to complete a checklist review form and are provided with free text boxes to elaborate on their assessment. These free text comments are reproduced below.

ARTICLE DETAILS

Title (Provisional)

Online Remote Behavioural Intervention for Tics (ORBIT-UK): Protocol of a single cohort usability study

Authors

Hastings, Olivia; Brown, Beverley J; Prentice, Kelly-Marie; Babbage, Camilla May; Davies, E Bethan; KILGARIFF, JOSEPH; Murphy, Tara; McGarry, Glenn; Guo, Boliang; Greenhalgh, Chris; Hollis, Chris; Hall, Charlotte Lucy

---

VERSION 1 - REVIEW

**Reviewer**	**1**
**Name**	**Rück, Christian**
**Affiliation**	**Karolinska Institutet, Department of Clinical Neuroscience**
**Date**	**24-Nov-2025**
**COI**	**None**

Thank you for the opportunity to review this study protocol for evaluating the ORBIT intervention within the NHS platform. The protocol is clearly written, addresses an important clinical need, and provides a strong rationale for examining how an academically developed intervention can be implemented within routine healthcare. The section on patient and public involvement is particularly well-developed and stands out as a strength of the manuscript.

The study has the potential to contribute valuable knowledge about scalable digital interventions for tic disorders, and the mixed-methods approach appears appropriate for the aims of the project. However, several methodological details require clarification, and some elements typically expected in a study protocol are only briefly described. Addressing these points would help strengthen the manuscript and improve its transparency.

Major comments

• Although the study is preregistered, the manuscript would benefit from more detail on the planned quantitative analysis. The preregistration would benefit from a statistical analysis

plan, and the manuscript should be more explicit about the statistical models to be used, and how missing data will be handled.

• The inclusion criteria “are clinically suitable for the intervention” could be described in more detail.

• Even though the dates are mentioned in the preregistration, please include the expected start and end dates for recruitment, follow-up, and overall study completion in the manuscript.

• It would also be helpful to specify and describe which mixed-methods approach will be carried out.

Minor comments and suggestions:

• As ORBIT builds on the BIP-TIC treatment model, the original publication should be cited to provide readers with relevant background (DOI: 10.1007/s10578-020-01098-y). Reference nr 10 may be pointing to the wrong paper.

• More clarification on the coaches’ clinical background would be helpful for readers.

• Inter-rater reliability testing of the YGTSS should either be conducted or a rationale provided for why it is not planned

• The CGI-I could be complemented by the CGI-S, with CGI-S assessed pretreatment and at the primary endpoint.

VERSION 1 - AUTHOR RESPONSE

Reviewer 1 Comments to the Author
Major comments
1) *Although the study is preregistered, the manuscript would benefit from more detail on the planned quantitative analysis. The preregistration would benefit from a statistical analysis plan, and the manuscript should be more explicit about the statistical models to be used, and how missing data will be handled.*

We have added additional detail to our quantitative analysis section of the manuscript on page 20. Specifically, we have clarified the descriptive statistics and regression models we plan to use, as well as how missing data will be handled. A statistical analysis plan has been added to the ISRTCN with full details of the quantitative analysis we plan to carry out.

2) *The inclusion criteria “are clinically suitable for the intervention” could be described in more detail.*

Thank you for this helpful comment. We agree that the inclusion criterion “clinical suitability for ORBIT confirmed by referrer from the patients’ usual care team” required further clarification. We have now expanded this description in the manuscript to explain that “clinical suitability” refers to the referring clinician’s professional judgement that ORBIT is an appropriate therapeutic option for the young person, based on their clinical presentation, motivation to engage, and capacity to undertake the required practice tasks. This aligns with routine real-world clinical practice, where

clinicians recommend interventions that they believe are likely to be beneficial and acceptable to the patient. We have updated the inclusion criteria section accordingly on page 10.

3) *Even though the dates are mentioned in the preregistration, please include the expected start and end dates for recruitment, follow-up, and overall study completion in the manuscript.*

We appreciate the reviewer noticing this missed detail. We have added the following information to the study design within the methods section of the manuscript on page 8. The planned recruitment start date is January 1st 2025, with an estimated end date of 21st September 2026. Overall study completion will be 3rd March 2027, and findings will be published within 1 year of data collection. Post-intervention measures will be completed within 2 weeks of finishing the intervention and there is no further follow-up.

4) *It would also be helpful to specify and describe which mixed-methods approach will be carried out.*

We agree this isn't the most accurate reflection of our approach and have removed this from the manuscript. We have updated the data management and analysis section of the manuscript on page 20 to clarify which quantitative and qualitative methods we plan to use.

Minor comments and suggestions

1) *As ORBIT builds on the BIP-TIC treatment model, the original publication should be cited to provide readers with relevant background (DOI: 10.1007/s10578-020-01098-y). Reference nr 10 may be pointing to the wrong paper.*

We have amended the citation so that it now points readers to the relevant paper on BIP-TIC on page 6 of the manuscript.

2) *More clarification on the coaches' clinical background would be helpful for readers.*

We agree that more information on the coaches' background would be helpful and have added detail on page 12. We clarify that the primary coach will be employed by Nottinghamshire Healthcare Foundation Trust (NHCFT), and the secondary coach will be a research assistant employed by the University of Nottingham, they will work within NHCFT under the terms of an honorary contract. Each coach will be fully trained in administering the YGTSS, trained in good clinical practice, and will be supervised throughout the study by the clinical team within NHCFT. Neither of the coaches have prior experience in delivering behavioural therapy for tic disorders.

Importantly, the coaches will not deliver the ORBIT intervention but simply support families as they work through the self-guided programme. Therefore, prior experience in delivering behavioural therapy is not essential. This reflects the intended real-world implementation of ORBIT, which aims to reduce reliance on highly specialised behavioural therapists to make treatment more accessible.

3) *Inter-rater reliability testing of the YGTSS should either be conducted or a rationale provided for why it is not planned*

We agree that this detail is missing and have added a section on page 16 of the tracked changes manuscript titled Agreement with Expert Rater (YGTSS). We explain that agreement with an expert rater will be assessed by the trial manager, an expert in the use of the YGTSS. The trial manager will independently score 10% of baseline and follow-up YGTSS assessments. They will observe coaches completing the YGTSS off camera with prior verbal consent and will not interact with families. When live observation is not possible, coaches will complete a standardised test assessment with the trial manager acting as a mock participant, followed by brief comparison and discussion of scoring.

Coaches must be within 15% of the trial manager's scores for the Total Motor Tic, Total Vocal Tic, and Total Tic Scores, following the approach described in previous research. Non-integer thresholds will be rounded up. Agreement calculations will be conducted by the statistician. Coaches who do not meet the threshold will receive further training and repeat scoring.

4) *The CGI-I could be complemented by the CGI-S, with CGI-S assessed pretreatment and at the primary endpoint.*

Thank you for this suggestion regarding the inclusion of the CGI-S. We appreciate the value of assessing severity alongside improvement. However, our outcome measures were intentionally aligned with those used in the previous ORBIT RCT, which employed the CGI-I only. For the present study, measure selection was guided by NHS clinicians and we opted for a more streamlined set of assessments that reflect routine clinical practice rather than a full clinical trial battery.
